# Supplementary material for: Renal UTX-PHGDH-serine axis regulates metabolic disorders in the kidney and liver
Source: Nat Commun. 2022 Jul 4;13:3835. doi: 10.1038/s41467-022-31476-0 (PMC9253056; doi:10.1038/s41467-022-31476-0)
Supplement: Supplementary file 1 — Supplemental Information [file 41467_2022_31476_MOESM1_ESM.pdf]

Supplemental Information

for

Renal UTX-PHGDH-Serine axis regulates metabolic disorders in the kidney and liver

Hong Chen[1], Chong Liu[2], Qian Wang[1], Mingrui Xiong[1], Xia Zeng[1], Dong Yang[1], Yunhao Xie[2], Hua Su[3], Yu Zhang[1], Yixue Huang[1], Yuchen Chen[1], Junqiu Yue[4], Chengyu Liu[5], Shun Wang[5], Kun Huang[1*] and Ling Zheng[2,6*]

[1]Tongji School of Pharmacy, Tongji Medical College, Huazhong University of Science and Technology, Wuhan, China, 430030

[2]Hubei Key Laboratory of Cell Homeostasis, College of Life Sciences, Wuhan University, Wuhan, China, 430072

[3]Department of Nephrology, Union Hospital, Tongji Medical College, Huazhong University of Science and Technology, Wuhan, China, 430030

[4]Department of Pathology, Hubei Cancer Hospital, Tongji Medical College, Huazhong University of Science and Technology, Wuhan, China, 430030.

[5]Department of Blood Transfusion, Wuhan Hospital of Traditional and Western Medicine, Wuhan, China, 430022

[6]Frontier Science Center for Immunology and Metabolism, Wuhan University, Wuhan, China, 430072

Corresponding authors

Ling Zheng, Ph.D.
College of Life Sciences
Wuhan University
Wuhan, China 430072
lzheng@whu.edu.cn

Kun Huang, Ph.D.
Tongji School of Pharmacy
Huazhong Univ. of Sci. & Tech.
Wuhan, China, 430030
kunhuang@hust.edu.cn

This supplementary file contains 22 Supplementary Figures and 11 Supplementary Tables.

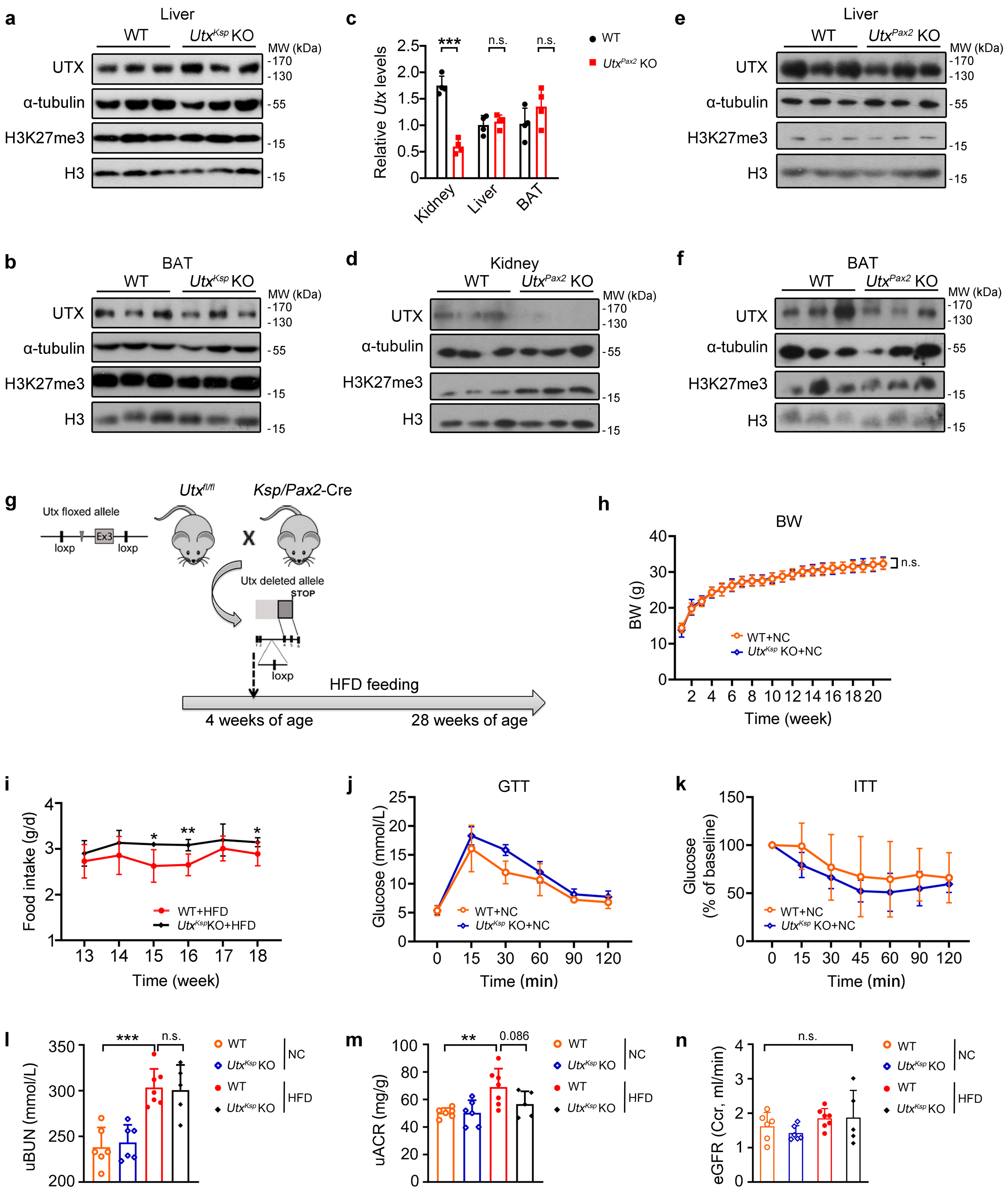

Supplementary Fig. 1 ***Utx**^{Ksp}* **KO mice showed no obvious phenotype under normal chow-fed conditions, and showed no obvious effect on food intake and renal functions**

under HFD-fed conditions. a-b, Western blot analysis of UTX levels in the liver (**a**) and BAT (**b**) of male WT and Utx^{Ksp} KO mice, n = 6 independent animals. **c-f**, qPCR (**c**) and Western blot analysis (**d-f**) of UTX levels in the kidney (**d**), liver (**e**) and BAT (**f**) of male WT and Utx^{Pax2} KO mice. c, n = 4 independent animals (mean ± SD), $^{***}P_{kidney} < 0.0001$ (unpaired, two-tailed t-test); d-f, n = 3 independent animals. **g**, Experimental design for breeding and HFD feeding. **h-i**, Growth curves (**h**) and food intake (**i**) of indicated groups. h, n = 6 independent animals; i, n = 8 or 6 independent animals for WT+HFD or Utx^{Ksp} KO +HFD group, respectively (mean ± SD, unpaired, two-tailed t-test). $^{*}P_{15\text{-}week} = 0.0406$, $^{**}P_{16\text{-}week} = 0.0011$, $^{*}P_{18\text{-}week} = 0.0294$ (unpaired, two-tailed t-test). **j-k**, Glucose tolerance test (GTT; **j**) and insulin tolerance test (ITT; **k**) results of WT and Utx^{Ksp} KO mice fed with normal chow, j, n =3 independent animals; k, n = 6 independent animals (mean ± SD). **l-n**, urine blood urea nitrogen (uBUN; **l**), urine albumin to creatinine ratios (uACR; **m**) and estimated glomerular filtration rate (eGFR; **n**) levels of indicated groups, l-n, n = 6, 6, 7, 5 independent animals for WT+NC or Utx^{Ksp} KO+NC or WT+HFD or Utx^{Ksp} KO +HFD group, respectively (mean ± SD). $^{***}P_{uBUN\ (WT+NC\ vs\ WT+HFD)} < 0.0001$; $^{**}P_{uACR\ (WT+NC\ vs\ WT+HFD)} = 0.0069$ (unpaired, two-tailed t-test). WT+NC, wild-type mice fed with normal chow; Utx^{Ksp} KO+NC, Utx^{Ksp} KO mice fed with normal chow; WT+HFD, wild-type mice fed with high fat diet; Utx^{Ksp} KO +HFD, Utx^{Ksp} KO mice fed with high fat diet. Source data are provided in the Source Data file.

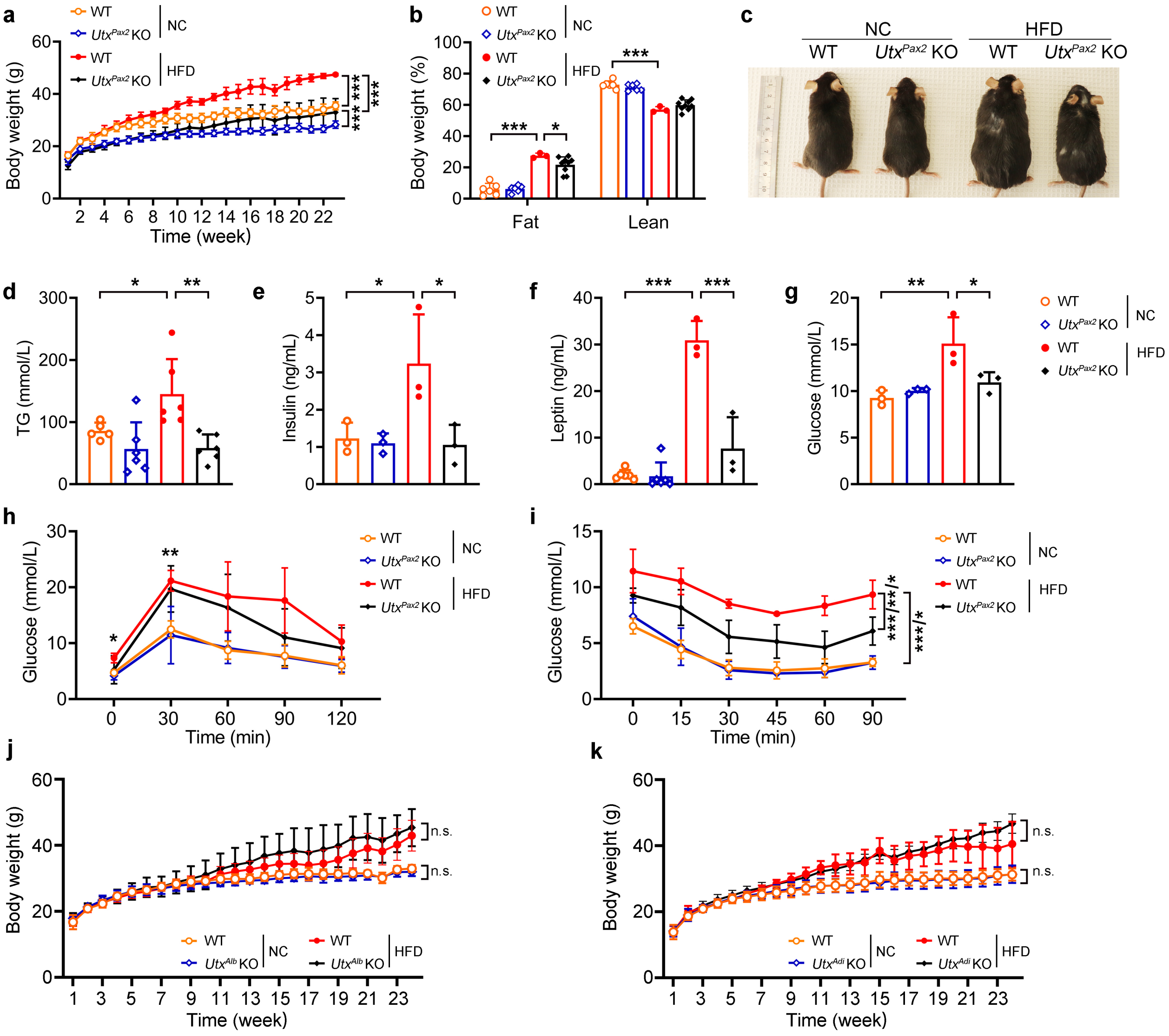

Supplementary Fig. 2 Reduced body weight, fat mass, blood insulin, leptin and glucose levels in *Utx^{Pax2}* KO male mice fed with HFD, and no obvious phenotype was observed in NC- or HFD-fed *Utx^{Alb}* KO or *Utx^{Adi}* KO male mice. a, Growth curves of WT and *Utx^{Pax2}* KO mice fed with NC or HFD, n = 6, 6, 3, 9 independent animals for WT+NC or *Utx^{Pax2}* KO+NC or WT+HFD or *Utx ^{Pax2}* KO +HFD group, respectively (mean ± SD). $^{***}P_{body\ weight\ (WT+NC\ vs\ WT+HFD)} < 0.0001$, $^{***}P_{body\ weight\ (WT+NC\ vs\ Utx^{Pax2}\ KO+NC)} < 0.0001$, $^{***}P_{body\ weight\ (WT+HFD\ vs\ Utx^{Pax2}\ KO+HFD)} < 0.0001$ (one-way ANOVA). **b**, Body composition, n = 6, 6, 3, 9 independent animals for WT+NC or *Utx^{Pax2}* KO+NC or WT+HFD or *Utx ^{Pax2}* KO +HFD group, respectively (mean ± SD). $^{***}P_{Fat\ (WT+NC\ vs\ WT+HFD)} < 0.0001$, $^{*}P_{Fat\ (WT+HFD\ vs\ Utx^{Pax2}\ KO+HFD)} =$

0.0125; $^{***}P_{Lean\ (WT+NC\ vs\ WT+HFD)} < 0.0001$ (one-way ANOVA). **c**, Abdominal view of WT and *Utx*^{Pax2} KO mice under NC or HFD conditions. **d-g**, Blood TG, insulin, leptin and glucose levels of WT and *Utx*^{Pax2} KO mice fed with NC or HFD. d, n = 5, 6, 6, 6 independent animals for WT+NC or *Utx*^{Pax2} KO+NC or WT+HFD or *Utx*^{Pax2} KO +HFD group, respectively; e, n = 3 independent animals; f, n = 6, 6, 3, 3 independent animals for WT+NC or *Utx*^{Pax2} KO+NC or WT+HFD or *Utx*^{Pax2} KO +HFD group, respectively; g, n = 3 independent animals (mean ± SD). $^{*}P_{TG\ (WT+NC\ vs\ WT+HFD)} = 0.0493$, $^{**}P_{TG\ (WT+HFD\ vs\ Utx^{Pax2}\ KO+HFD)} = 0.0026$; $^{*}P_{Insulin\ (WT+NC\ vs\ WT+HFD)} = 0.0287$, $^{*}P_{Insulin\ (WT+HFD\ vs\ Utx^{Pax2}\ KO+HFD)} = 0.0192$; $^{***}P_{Leptin\ (WT+NC\ vs\ WT+HFD)} < 0.0001$, $^{***}P_{Leptin\ (WT+HFD\ vs\ Utx^{Pax2}\ KO+HFD)} < 0.0001$; $^{**}P_{Glucose\ (WT+NC\ vs\ WT+HFD)} = 0.0048$, $^{*}P_{Glucose\ (WT+HFD\ vs\ Utx^{Pax2}\ KO+HFD)} = 0.0288$ (one-way ANOVA). **h-i**, Glucose tolerance test (GTT) and insulin tolerance test (ITT) results of the indicated groups. h, n = 3, 3, 3, 6 independent animals for WT+NC or *Utx*^{Pax2} KO+NC or WT+HFD or *Utx*^{Pax2} KO +HFD group, respectively; i, n = 5, 5, 4, 6 independent animals for WT+NC or *Utx*^{Pax2} KO+NC or WT+HFD or *Utx*^{Pax2} KO +HFD group, respectively (mean ± SD). $^{*}P_{GTT\ (0,\ WT+NC\ vs\ WT+HFD)} = 0.0171$, $^{**}P_{GTT\ (30,\ WT+NC\ vs\ WT+HFD)} = 0.0037$; $^{*}P_{ITT\ (0,\ WT+NC\ vs\ WT+HFD)} = 0.0108$, $^{***}P_{ITT\ (15,\ WT+NC\ vs\ WT+HFD)} = 0.0003$, $^{***}P_{ITT\ (30,\ WT+NC\ vs\ WT+HFD)} < 0.0001$, $^{***}P_{ITT\ (45,\ WT+NC\ vs\ WT+HFD)} < 0.0001$, $^{***}P_{ITT\ (60,\ WT+NC\ vs\ WT+HFD)} < 0.0001$, $^{***}P_{ITT\ (90,\ WT+NC\ vs\ WT+HFD)} < 0.0001$, $^{*}P_{ITT\ (15,\ WT+HFD\ vs\ Utx^{Pax2}\ KO+HFD)} = 0.0292$, $^{**}P_{ITT\ (30,\ WT+HFD\ vs\ Utx^{Pax2}\ KO+HFD)} = 0.0035$, $^{**}P_{ITT\ (45,\ WT+HFD\ vs\ Utx^{Pax2}\ KO+HFD)} = 0.0095$, $^{***}P_{ITT\ (60,\ WT+HFD\ vs\ Utx^{Pax2}\ KO+HFD)} = 0.0009$, $^{**}P_{ITT\ (90,\ WT+HFD\ vs\ Utx^{Pax2}\ KO+HFD)} = 0.0061$ (one-way ANOVA). **j-k**, Growth curves of WT and *Utx*^{Alb} KO or *Utx*^{Adi} KO mice fed with NC or HFD. j, n = 6, 5, 6, 6 independent animals for WT+NC or *Utx*^{Alb} KO+NC or WT+HFD or *Utx*^{Alb} KO +HFD group, respectively; k, n = 9, 9, 6, 6 independent animals for WT+NC or *Utx*^{Adi} KO+NC or WT+HFD or *Utx*^{Adi} KO +HFD group, respectively (mean ± SD, one-way ANOVA). WT+NC, wild-type mice fed with normal chow; *Utx*^{Pax2}/*Utx*^{Alb}/*Utx*^{Adi} KO+NC, *Utx*^{Pax2}/*Utx*^{Alb}/*Utx*^{Adi} KO mice fed with normal chow; WT+HFD, wild-type mice fed with high fat diet; *Utx*^{Pax2}/*Utx*^{Alb}/*Utx*^{Adi} KO +HFD, *Utx*^{Pax2}/*Utx*^{Alb}/*Utx*^{Adi} KO mice fed with high fat diet. Source data are provided in the Source Data file.

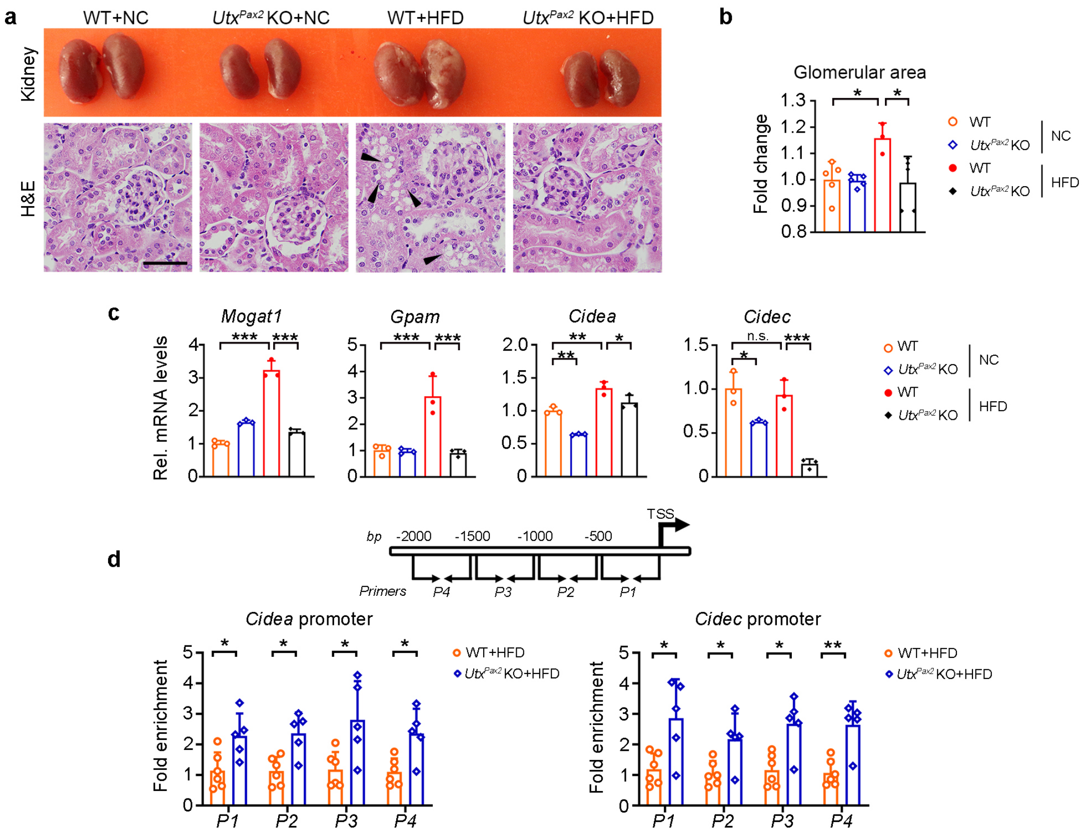

Supplementary Fig. 3 HFD-fed *Utx^{Pax2}* KO mice showed reduced lipid accumulation in the kidney. a-b, Representative kidneys and representative images of H&E staining in the WT and *Utx^{Pax2}* KO mice fed with NC or HFD (**a**), with quantitative data for relative glomerular area (**b**), n = 5, 5, 3, 5 independent animals for WT+NC or *Utx^{Pax2}* KO+NC or WT+HFD or *Utx^{Pax2}* KO +HFD group, respectively (mean ± SD). $^{*}P_{H\&E\ (WT+NC\ vs\ WT+HFD)}$ = 0.0182, $^{*}P_{H\&E\ (WT+HFD\ vs\ Utx^{Pax2}\ KO+HFD)}$ = 0.012 (one-way ANOVA). Scale bar, 50 µm. **c**, qPCR analysis for TG synthesis and storage related genes in the indicated groups, n = 3 independent animals (mean ± SD). $^{***}P_{Mogat1\ (WT+NC\ vs\ WT+HFD)}$ < 0.0001, $^{***}P_{Mogat1\ (WT+HFD\ vs\ Utx^{Pax2}\ KO+HFD)}$ < 0.0001; $^{***}P_{Gpam\ (WT+NC\ vs\ WT+HFD)}$ = 0.0007, $^{***}P_{Gpam\ (WT+HFD\ vs\ Utx^{Pax2}\ KO+HFD)}$ = 0.0005; $^{**}P_{Cidea\ (WT+NC\ vs\ Utx^{Pax2}\ KO+NC)}$ = 0.0021, $^{**}P_{Cidea\ (WT+NC\ vs\ WT+HFD)}$ = 0.0031, $^{*}P_{Cidea\ (WT+HFD\ vs\ Utx^{Pax2}\ KO+HFD)}$ = 0.0385; $^{*}P_{Cidec\ (WT+NC\ vs\ Utx^{Pax2}\ KO+NC)}$ = 0.0281, $^{***}P_{Cidec\ (WT+HFD\ vs\ Utx^{Pax2}\ KO+HFD)}$ = 0.0003, (one-way ANOVA). **d**, ChIP assay for H3K27me3 on the promoters of *Cidea* and *Cidec* in indicated groups, n = 6, 5 independent animals for

WT+HFD or *Utx* ^{Pax2} KO +HFD group, respectively (mean ± SD). $^{*}P_{Cidea\ P1}$ = 0.0244, $^{*}P_{Cidea\ P2}$ = 0.0113; $^{*}P_{Cidea\ P3}$ = 0.0424, $^{*}P_{Cidea\ P4}$ = 0.0212; $^{*}P_{Cidec\ P1}$ = 0.0394, $^{*}P_{Cidec\ P2}$ = 0.0376, $^{*}P_{Cidec\ P3}$ = 0.0152, $^{**}P_{Cidec\ P4}$ = 0.006 (unpaired, two-tailed t-test). TSS, transcription start site. WT+NC, wild-type mice fed with normal chow; *Utx*^{Pax2} KO+NC, *Utx*^{Pax2} KO mice fed with normal chow; WT+HFD, wild-type mice fed with high fat diet; *Utx*^{Pax2} KO+HFD, *Utx*^{Pax2} KO mice fed with high fat diet. Source data are provided in the Source Data file.

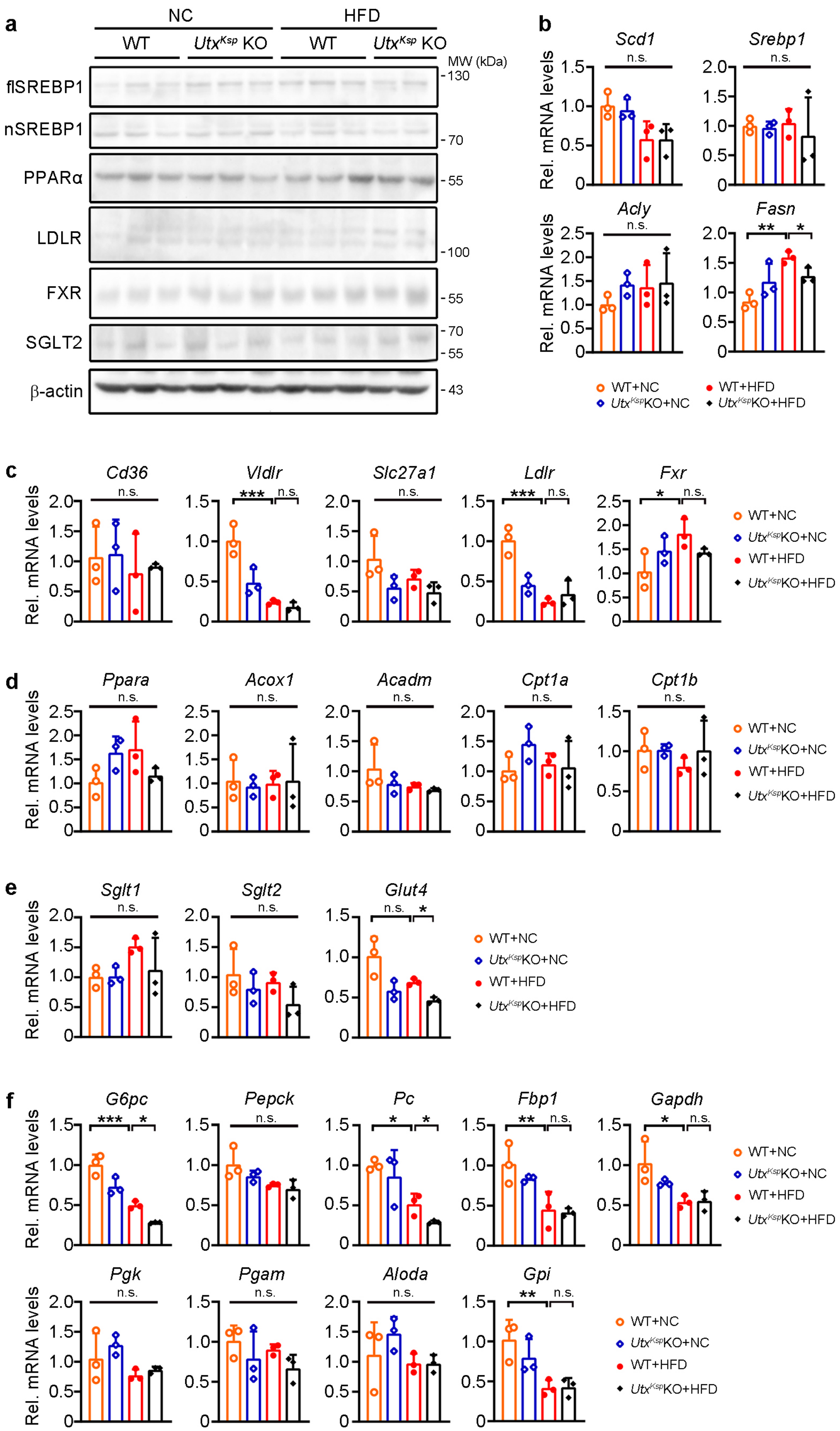
a
NC
HFD
WT
UtxKsp KO
WT
UtxKsp KO
MW (kDa)
flSREBP1
nSREBP1
PPARα
LDLR
FXR
SGLT2
β-actin
130
70
55
100
55
70
55
43
b
Scd1
Srebp1
Acly
Fasn
Rel. mRNA levels
n.s.
WT+NC
UtxKspKO+NC
WT+HFD
UtxKspKO+HFD
c
Cd36
Vldlr
Slc27a1
Ldlr
Fxr
d
Ppara
Acox1
Acadm
Cpt1a
Cpt1b
e
Sglt1
Sglt2
Glut4
f
G6pc
Pepck
Pc
Fbp1
Gapdh
Pgk
Pgam
Aloda
Gpi

Supplementary Fig. 4 HFD-fed *Utx*^{ksp} KO mice showed mild effects on glucose and lipid metabolic pathways in the renal cortex. a, Western blot analysis of SREBP1, PPARα, FXR, LDLR and SGLT2 in the WT and *Utx*^{Ksp} KO mice under NC- and HFD-fed conditions, n = 3, 3, 3, 2 independent animals for WT+NC or *Utx*^{Ksp} KO+NC or WT+HFD or *Utx*^{Ksp} KO +HFD group, respectively. **b-f**, qPCR results of genes involved in lipid synthesis (**b**), lipid transport (**c**), beta oxidation (**d**), glucose transport (**e**), glyconeogenesis and glycolysis pathways (**f**) in the WT and *Utx*^{Ksp} KO mice under NC- and HFD-fed conditions, n = 3 independent animals (mean ± SD). $^{**}P_{Fasn\ (WT+NC\ vs\ WT+HFD)} = 0.0033$, $^{*}P_{Fasn\ (WT+HFD\ vs\ Utx^{Ksp}\ KO+HFD)} = 0.0401$; $^{***}P_{Vldlr\ (WT+NC\ vs\ WT+HFD)} = 0.0003$; $^{***}P_{Ldlr\ (WT+NC\ vs\ WT+HFD)} = 0.0003$; $^{*}P_{Fxr\ (WT+NC\ vs\ WT+HFD)} = 0.0249$; $^{*}P_{Glut4\ (WT+HFD\ vs\ Utx^{Ksp}\ KO+HFD)} = 0.0454$; $^{***}P_{G6pc\ (WT+NC\ vs\ WT+HFD)} = 0.0003$; $^{*}P_{G6pc\ (WT+HFD\ vs\ Utx^{Ksp}\ KO+HFD)} = 0.039$; $^{*}P_{Pc\ (WT+NC\ vs\ WT+HFD)} = 0.0277$, $^{*}P_{Pc\ (WT+HFD\ vs\ Utx^{Ksp}\ KO+HFD)} = 0.0495$; $^{**}P_{Fbp1\ (WT+NC\ vs\ WT+HFD)} = 0.0091$; $^{*}P_{Gapdh\ (WT+NC\ vs\ WT+HFD)} = 0.0124$; $^{**}P_{Gpi\ (WT+NC\ vs\ WT+HFD)} = 0.0099$ (one-way ANOVA). Source data are provided in the Source Data file.

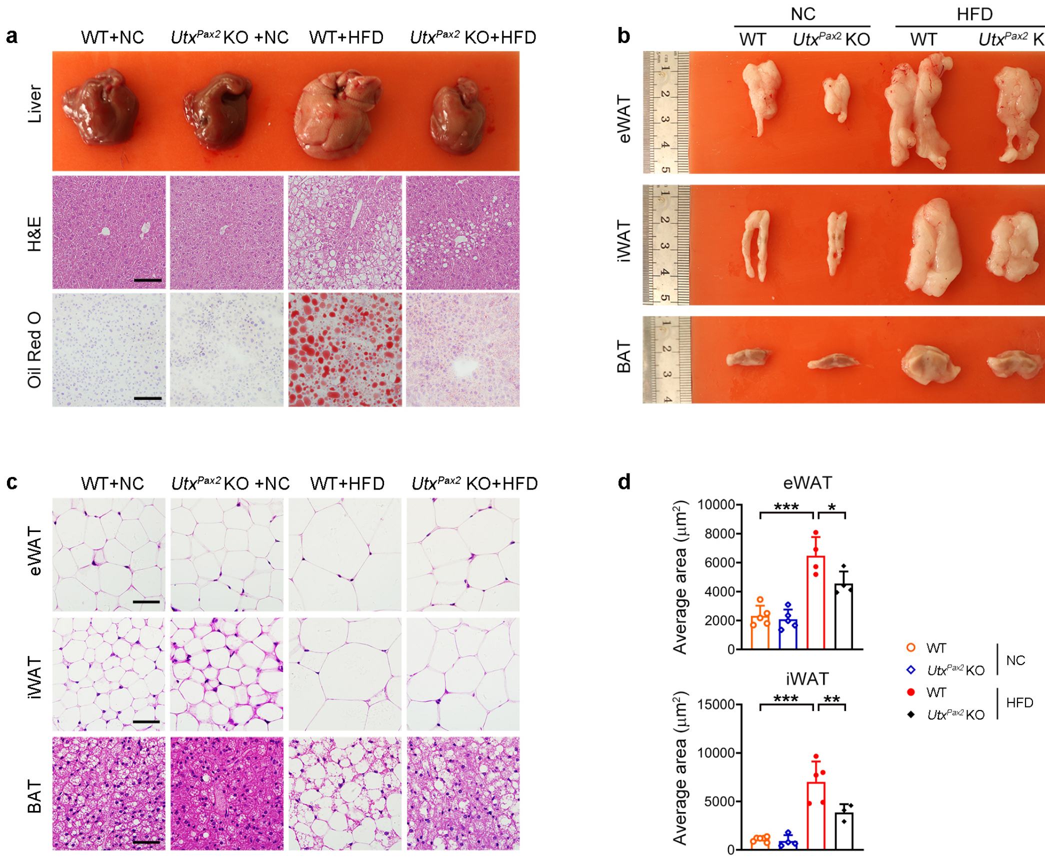

Supplementary Fig. 5 HFD-fed *Utx*^{Pax2} KO mice show reduced lipid accumulation in the liver and adipose tissues. a, Representative images of livers and images of H&E and Oil Red O staining in the liver tissue of WT and *Utx*^{Pax2} KO mice fed with NC or HFD, n = 3-6 independent animals. **b**, Representative images of different fat tissues, n = 3-6 independent animals. **c-d**, H&E staining and average cell area in indicated groups, n_{eWAT} = 5, 5, 4, 4 independent animals for WT+NC or *Utx*^{Pax2} KO+NC or WT+HFD or *Utx* ^{Pax2} KO +HFD group, respectively; n_{iWAT} = 6, 4, 5, 3 independent animals for WT+NC or *Utx*^{Pax2} KO+NC or WT+HFD or *Utx* ^{Pax2} KO +HFD group, respectively, data shown as mean ± SD. $^{***}P_{eWAT\ (WT+NC\ vs\ WT+HFD)} < 0.0001$, $^{*}P_{eWAT\ (WT+HFD\ vs\ Utx^{Pax2}\ KO+HFD)} = 0.0212$; $^{***}P_{iWAT\ (WT+NC\ vs\ WT+HFD)} < 0.0001$, $^{**}P_{iWAT\ (WT+HFD\ vs\ Utx^{Pax2}\ KO+HFD)} = 0.0087$ (one-way ANOVA). WT+NC, wild-type mice fed with normal chow; *Utx*^{Pax2} KO+NC, *Utx*^{Pax2} KO mice fed with normal chow; WT+HFD, wild-type mice fed with high fat diet; *Utx*^{Pax2} KO+HFD, *Utx*^{Pax2} KO mice fed with high fat diet. Source data are provided in the Source Data file.

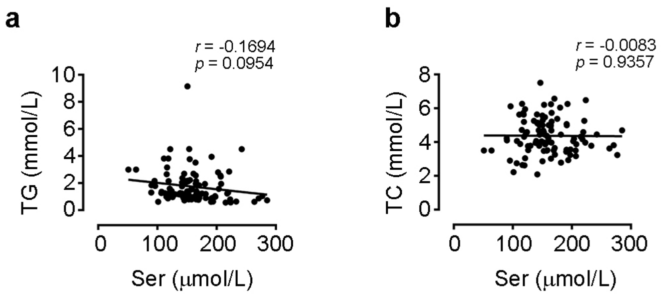

Supplementary Fig. 6 Correlation between serum serine level and triglycerides or cholesterol. Correlation analysis between the serum serine level and serum triglycerides (TG; **a**), or serum cholesterol (TC; **b**) from individuals with normal body weight (n = 39), overweight (n = 23), obesity (n = 24) or obesity-related renal dysfunction (n = 12). Correlation analysis was performed by Pearson's method. The Pearson correlation coefficients and *p* values (two-tailed test) are shown. Source data are provided in the Source Data file.

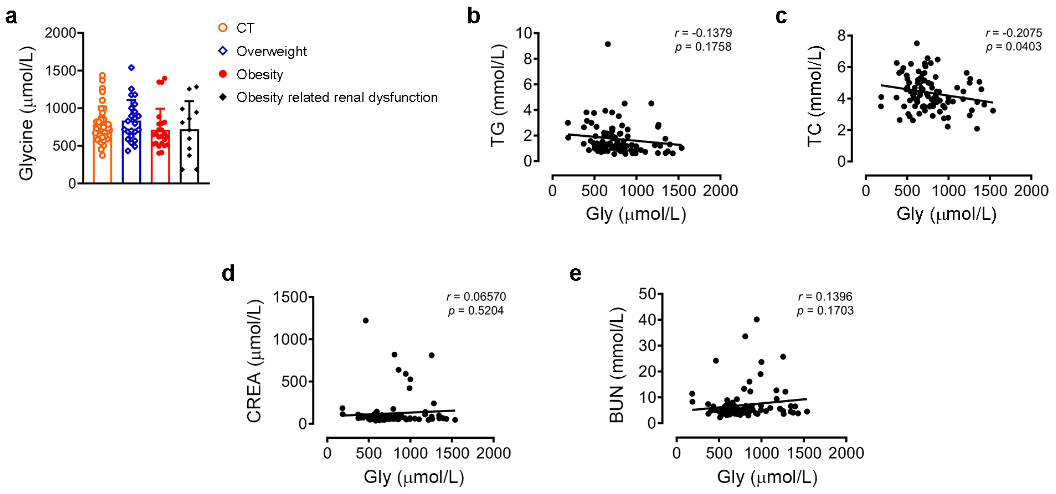

Supplementary Fig. 7 Correlation between serum glycine level and different parameters. **a**, Serum glycine level of indicated groups. Data shown as mean ± SD. **b**-**e**, Correlation analysis between the serum glycine level and serum triglycerides (TG**; b**), cholesterol (TC; **c**), creatinine (CREA; **d**) and BUN (blood urea nitrogen; **e**) level from individuals with normal body weight (n = 39), overweight (n = 23), obesity (n = 24) or obesity-related renal dysfunction (n = 12). Correlation analysis was performed by Pearson's method. The Pearson correlation coefficients and *p* values (two-tailed test) are shown. Source data are provided in the Source Data file.

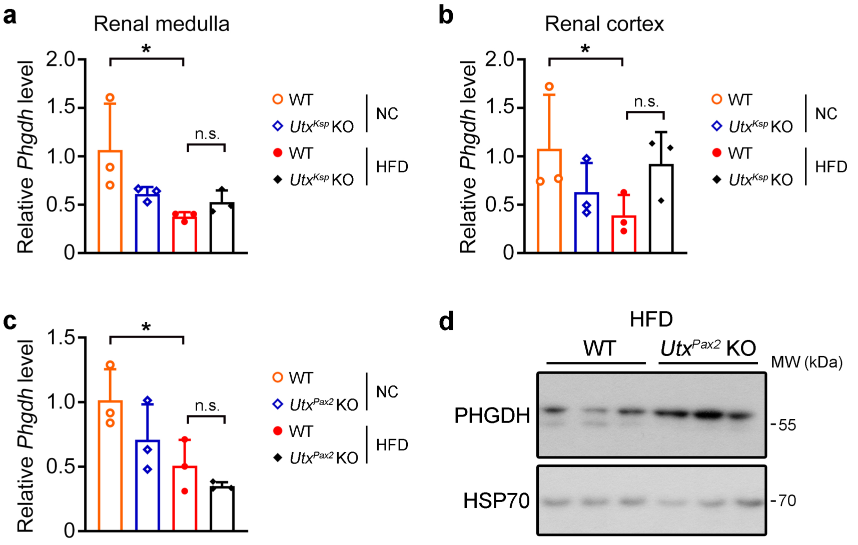

Supplementary Fig. 8 *Utx* knockout showed no effect on *Phgdh* mRNA level but a significant increase of its protein level in the kidneys of *Utx*^{Pax2} KO mice under HFD stress. qPCR analysis of *Phdgh* levels in the renal medulla (**a**) and cortex (**b**) of WT and *Utx*^{Ksp} KO mice, and in the kidneys of WT and *Utx*^{Pax2} KO mice (**c**) under NC or HFD conditions, n = 3 independent animals (mean ± SD). (a)$^{*}P_{(WT+NC\ vs\ WT+HFD)}$ = 0.0244; (b)$^{*}P_{(WT+NC\ vs\ WT+HFD)}$ = 0.0495; (c)$^{*}P_{(WT+NC\ vs\ WT+HFD)}$ = 0.0435 (one-way ANOVA). **d**, PHGDH level in the kidney of WT and *Utx*^{Pax2} KO mice fed with HFD, n = 3 independent animals. WT+NC, wild-type mice fed with normal chow; *Utx*$^{Ksp/Pax2}$ KO+NC, *Utx*$^{Ksp/Pax2}$ KO mice fed with normal chow; WT+HFD, wild-type mice fed with high fat diet; *Utx*$^{Ksp/Pax2}$ KO +HFD, *Utx*$^{Ksp/Pax2}$ KO mice fed with high fat diet. Source data are provided in the Source Data file.

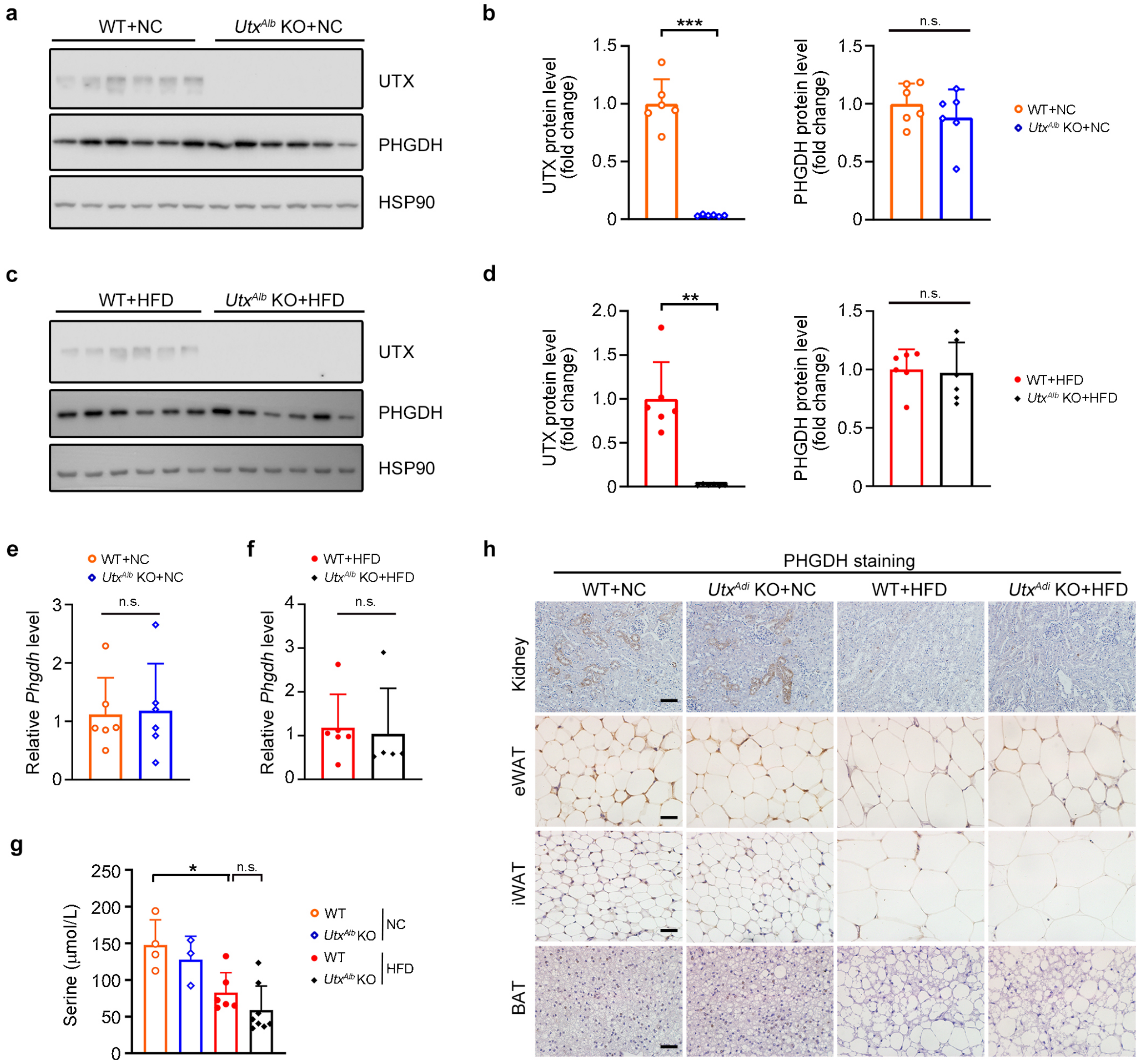

Supplementary Fig. 9 Liver or adipose tissue specific knockout *Utx* showed no effect on PHGDH or serine levels in male mice. a-d, Representative Western blots and densitometric quantitative results of UTX, and PHGDH in the liver of WT and Utx^{Alb} KO mice under NC (**a-b**) or HFD (**c-d**) conditions, n = 6 independent animals (mean ± SD). (b) $^{***}P_{UTX}$ = 0.0001; (d) $^{**}P_{UTX}$ = 0.0022 (unpaired, two-tailed t-test). **e-f**, qPCR analysis of *Phdgh* levels in the liver of WT and Utx^{Alb} KO mice under NC (**e**) or HFD (**f**) conditions, n = 6 independent animals (mean ± SD, unpaired, two-tailed t-test). **g**, Serum serine level of indicated groups, n = 4, 3, 6, 8 independent animals for WT+NC or Utx^{Alb} KO+NC or WT+HFD or Utx^{Alb} KO +HFD group, respectively (mean ± SD). $^{*}P_{serine\ (WT+NC\ vs\ WT+HFD)}$ = 0.0144 (one-way ANOVA). **h**,

Representative images of PHGDH staining in WT and *Utx*^{Adi} KO mice under NC or HFD conditions, n = 3-6 independent animals. Scale bar, 50 μm. Source data are provided in the Source Data file.

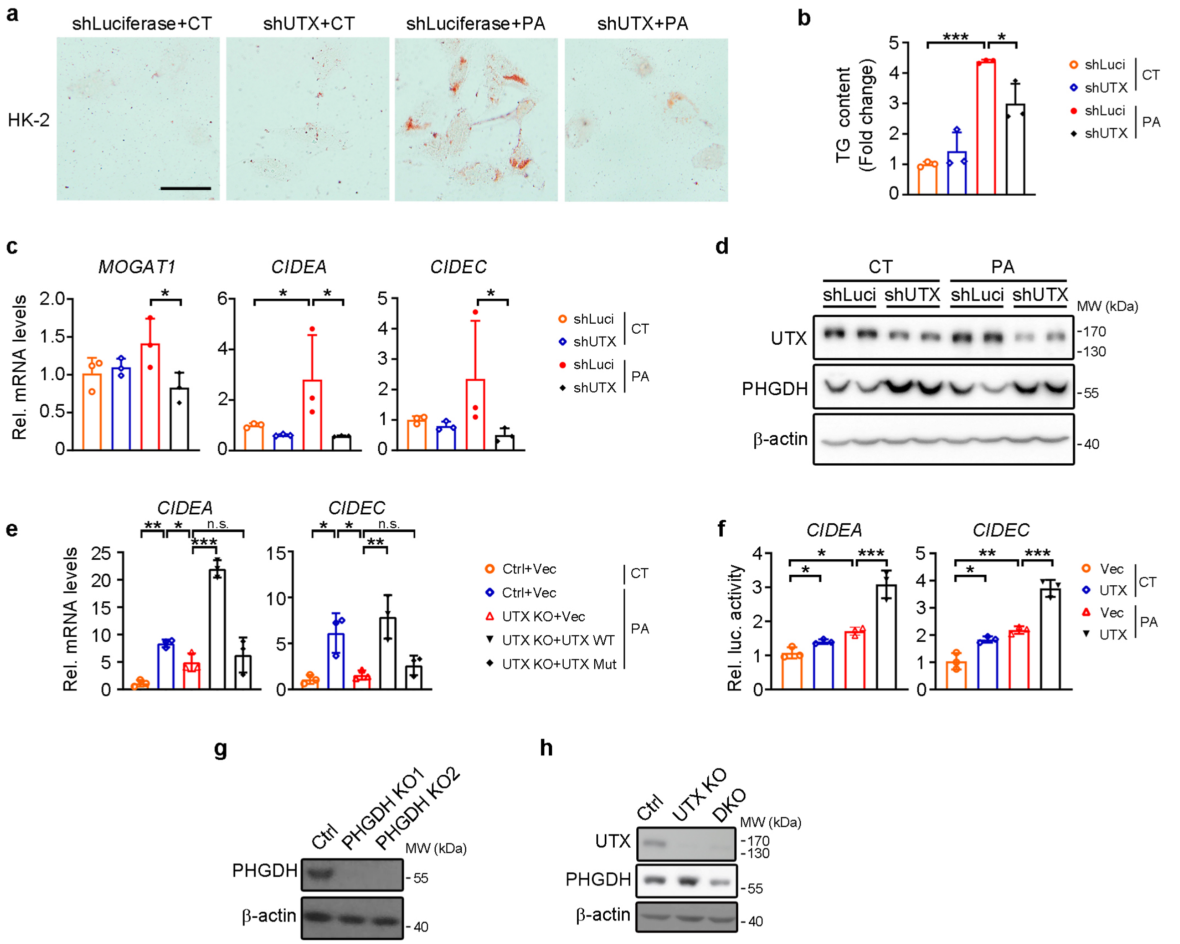

Supplementary Fig. 10 UTX regulated lipid accumulation in HK-2 cells. Representative images of Oil Red O staining (**a**) and TG concentrations (**b**) in HK-2 cells with or without UTX knockdown, n = 3 biological samples per group (mean ± SD). $^{***}P_{TG\ (shLuci+CT\ vs\ shLuci+PA)} < 0.0001$, $^{*}P_{TG\ (shLuci+PA\ vs\ shUTX+PA)} = 0.0142$ (one-way ANOVA). Scale bar, 50 μm. **c**, Transcriptional changes of TG synthesis/lipid storage related genes in indicated groups, n = 3 biological samples per group (mean ± SD). $^{*}P_{MOGAT1(shLuci+PA\ vs\ shUTX+PA)} = 0.0322$; $^{*}P_{CIDEA\ (shLuci+CT\ vs\ shLuci+PA)} = 0.0495$, $^{*}P_{CIDEA\ (shLuci+PA\ vs\ shUTX+PA)} = 0.036$; $^{*}P_{CIDEC\ (shLuci+PA\ vs\ shUTX+PA)} = 0.0495$ (one-way ANOVA). **d**, Western blot analysis of PHGDH levels in UTX knockdown HK-2 cells with or without PA treatment, n = 2 biological samples per group. **e**, Transcriptional levels of *CIDEA* and *CIDEC* of indicated groups, n = 3 biological samples per group (mean ± SD). $^{**}P_{CIDEA\ (Ctrl+Vec+CT\ vs\ Ctrl+Vec+PA)} = 0.0041$, $^{*}P_{CIDEA\ (Ctrl+Vec+PA\ vs\ UTX\ KO+Vec+PA)} = 0.0495$, $^{***}P_{CIDEA\ (UTX\ KO+Vec+PA\ vs\ UTX\ KO+UTX}$

$_{WT+PA)}$ < 0.0001; $^{*}P_{CIDEC\ (Ctrl+Vec+CT\ vs\ Ctrl+Vec+PA)}$ = 0.0163, $^{*}P_{CIDEC\ (Ctrl+Vec+PA\ vs\ UTX\ KO+Vec+PA)}$ = 0.0293, $^{**}P_{CIDEC\ (UTX\ KO+Vec+PA\ vs\ UTX\ KO+UTX\ WT+PA)}$ = 0.0036 (one-way ANOVA). **f**, Luciferase reporter assays that examine the effects of UTX on *CIDEA* and *CIDEC* promoters in HK-2 cells, n =3 biological samples per group (mean ± SD). $^{*}P_{CIDEA\ (Vec+CT\ vs\ UTX+CT)}$ = 0.0495, $^{*}P_{CIDEA\ (Vec+CT\ vs\ Vec+PA)}$ = 0.0379, $^{***}P_{CIDEA\ (Vec+PA\ vs\ UTX+PA)}$ = 0.0004; $^{*}P_{CIDEC\ (Vec+CT\ vs\ UTX+CT)}$ = 0.0134, $^{**}P_{CIDEC\ (Vec+CT\ vs\ Vec+PA)}$ = 0.0015, $^{***}P_{CIDEC\ (Vec+PA\ vs\ UTX+PA)}$ = 0.0002 (one-way ANOVA). **g**, Representative Western blot analysis of PHGDH level in PHGDH knockout HK-2 cells. **h**, Representative Western blot analysis of UTX and PHGDH levels in UTX knockout (UTX KO), UTX/PHGDH double knockout (DKO) HK-2 cells. a-f, at least 3 independent experiments were performed and similar results were obtained. Source data are provided in the Source Data file.

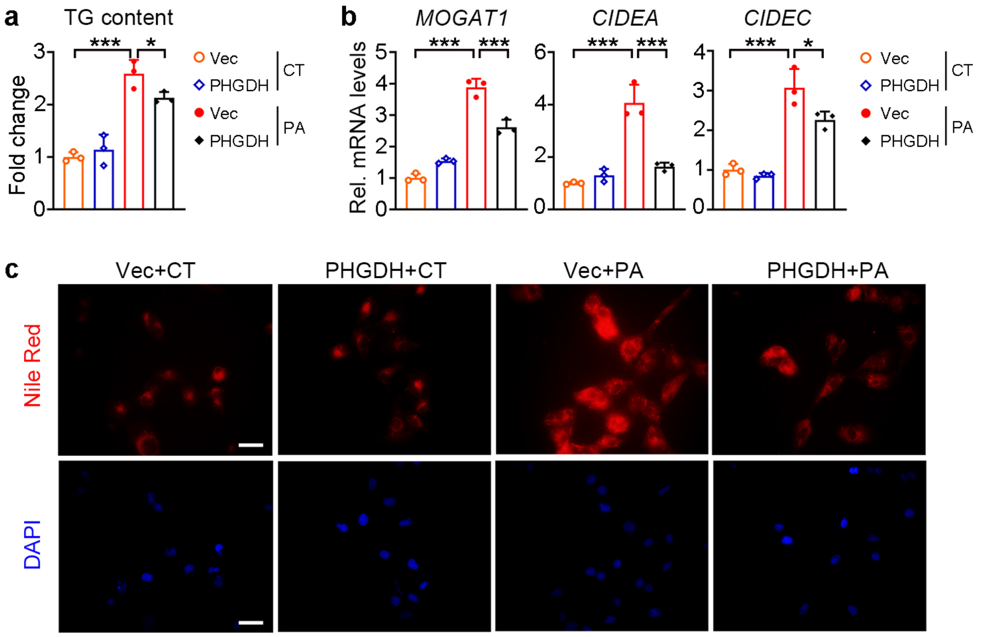

Supplementary Fig. 11 PHGDH overexpression inhibited lipid accumulation in palmitic acid stressed HK-2 cells. a-c, TG level (**a**), transcriptional levels of TG synthesis/storage related genes (**b**) and Nile Red staining (**c**) in PHGDH overexpressing HK-2 cells, n =3 biological samples per group (mean ± SD); at least 3 independent experiments were performed and similar results were obtained. $^{***}P_{TG\ (Vec+CT\ vs\ Vec+PA)} < 0.0001$, $^{*}P_{TG\ (Vec+PA\ vs\ PHGDH+PA)} = 0.0495$; $^{***}P_{MOGAT1\ (Vec+CT\ vs\ Vec+PA)} < 0.0001$, $^{***}P_{MOGAT1\ (Vec+PA\ vs\ PHGDH+PA)} = 0.0001$; $^{***}P_{CIDEA\ (Vec+CT\ vs\ Vec+PA)} < 0.0001$, $^{***}P_{CIDEA\ (Vec+PA\ vs\ PHGDH+PA)} = 0.0001$; $^{***}P_{CIDEC\ (Vec+CT\ vs\ Vec+PA)} < 0.0001$, $^{*}P_{CIDEC\ (Vec+PA\ vs\ PHGDH+PA)} = 0.016$ (one-way ANOVA). Scale bar, 50 μm. Source data are provided in the Source Data file.

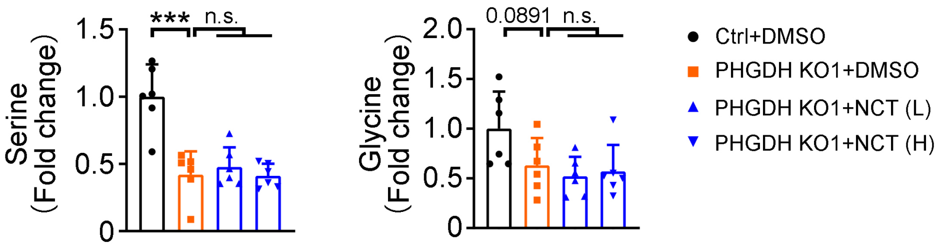

Supplementary Fig. 12 NCT-503 treatment showed no further effects on serine and glycine levels in PHGDH KO HK-2 cells. Serine (left) and glycine (right) levels in the PHGDH knockout or NCT-503 treated PHGDH knockout cells, n = 6 biological samples per group (mean ± SD); at least 3 independent experiments were performed and similar results were obtained. $^{***}P_{serine\ (Ctrl+DMSO\ vs\ PHGDH\ KO1+DMSO)} < 0.0001$ (one-way ANOVA). Source data are provided in the Source Data file.

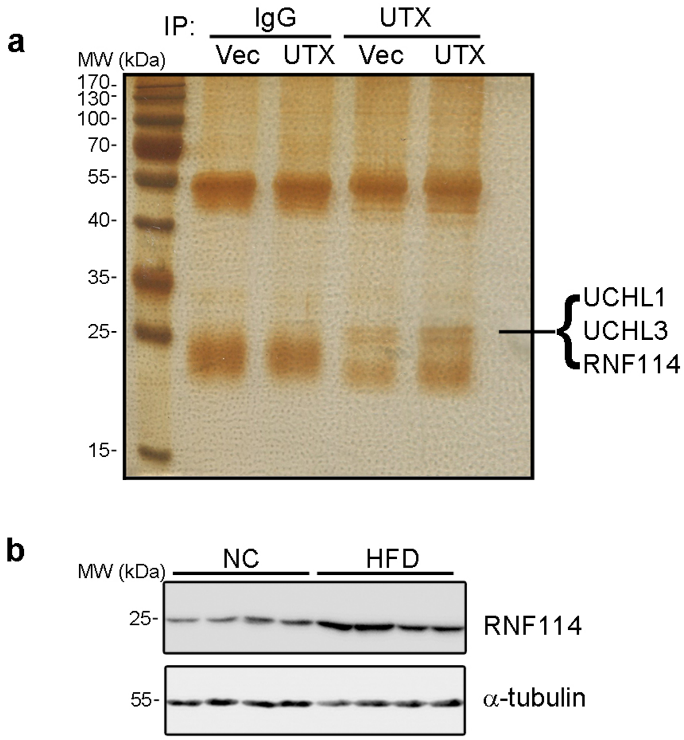

Supplementary Fig. 13 RNF114 was a possible binding partner of UTX that was increased upon HFD stress in the kidney. a, Silver staining and mass-spectrometry results of immunoprecipitation suggested possible binding partners of UTX**.** The SDS-PAGE and staining were repeated for three times. **b**, Representative Western blots of RNF114 in the kidney of WT mice under NC and HFD conditions, n = 6 independent animals. Source data are provided in the Source Data file.

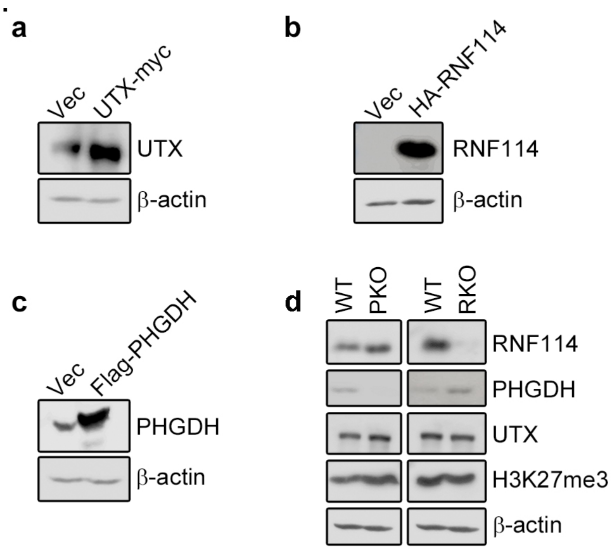

Supplementary Fig. 14 Transient overexpression of UTX, RNF114, and PHGDH in cultured cells. a-c, Western blot analysis of UTX, RNF114, PHGDH and β-actin levels in HEK293T cells when overexpressed UTX-myc (**a**), HA-RNF114 (**b**), or Flag-PHGDH (**c**). **d**, Western blot analysis of RNF114, PHGDH, UTX, H3K27me3, and β-actin levels in indicated HK-2 cells. PKO, PHGDH knockout HK-2 cells (PHGDH KO1); RKO, RNF114 knockout HK-2 cells. a-c**,** at least 2 independent experiments were performed to verify the expression efficiency of plasmids; d, 3 independent experiments were performed and similar results were obtained. Source data are provided in the Source Data file.

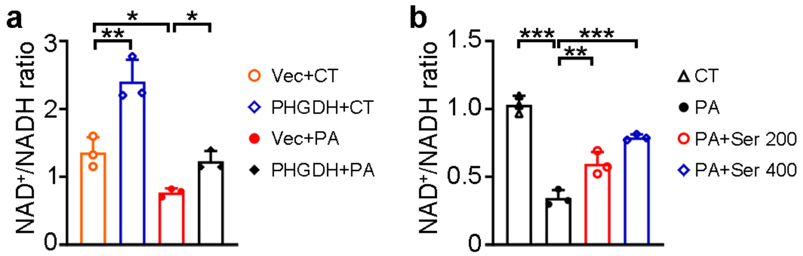

Supplementary Fig. 15 Overexpression of PHGDH or serine treatment increased NAD^+/NADH ratio in HK-2 cells. NAD^+/NADH ratio in the PHGDH overexpression (**a**) or serine-treated (**b**) HK-2 cells, n = 3 biological samples per group (mean ± SD); at least 3 independent experiments were performed and similar results were obtained. (a) $^{**}P_{NAD^+/NADH\ (Vec+CT\ vs\ PHGDH+CT)} = 0.0014$, $^{*}P_{NAD^+/NADH\ (Vec+CT\ vs\ Vec+PA)} = 0.0378$, $^{*}P_{NAD^+/NADH\ (Vec+PA\ vs\ PHGDH+PA)} = 0.0208$; (b) $^{***}P_{NAD^+/NADH\ (CT\ vs\ PA)} < 0.0001$, $^{**}P_{NAD^+/NADH\ (PA\ vs\ PA+Ser200)} = 0.0031$, $^{***}P_{NAD^+/NADH\ (PA\ vs\ PA+Ser400)} < 0.0001$ (one-way ANOVA). Source data are provided in the Source Data file.

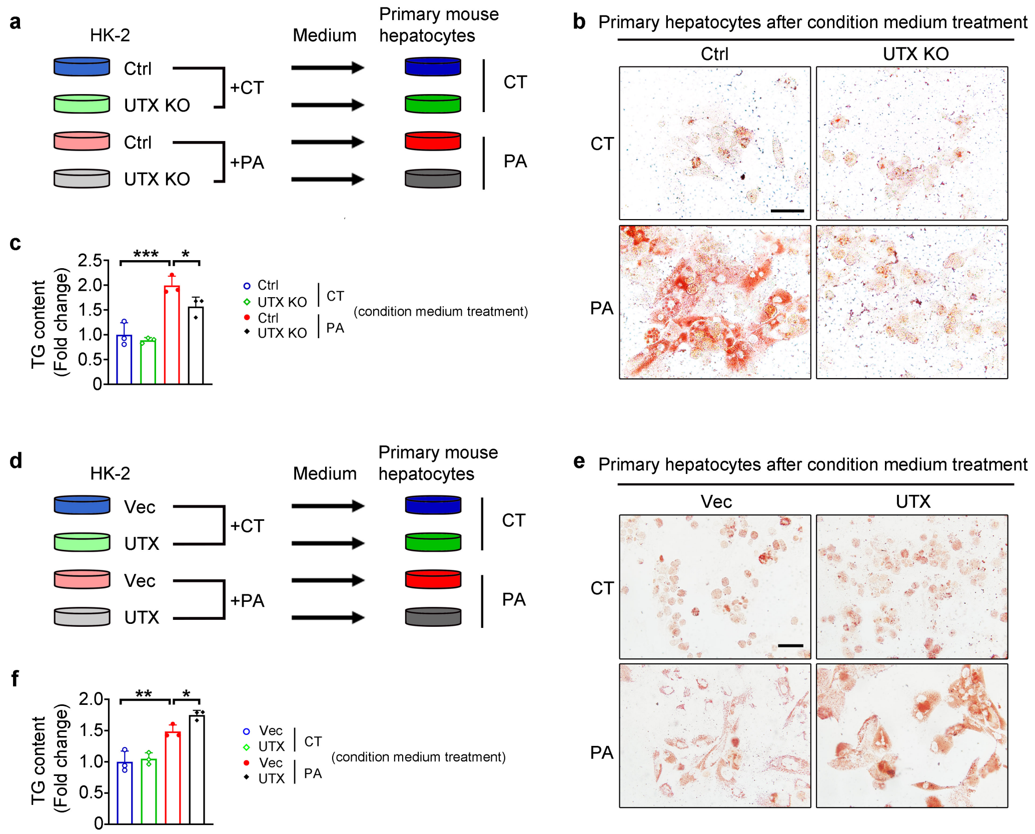

Supplementary Fig. 16 Conditional medium from UTX knockout or overexpressed HK-2 cells altered lipid accumulation in palmitic acid treated primary mouse hepatocytes. a-c, Experiment design (**a**), Oil Red O staining (**b**) and TG concentration (**c**) of primary mouse hepatocytes treated by the medium from the indicated groups. Scale bar, 100 μm, n = 3 biological samples per group (mean ± SD); at least 3 independent experiments were performed and similar results were obtained. Images were taken under a Sunny RX50 microscope. $^{***}P_{TG\ (Ctrl+CT\ vs\ Ctrl+PA)} = 0.0004$, $^{*}P_{TG\ (Ctrl+PA\ vs\ UTX\ KO+PA)} = 0.0495$ (one-way ANOVA). **d-f**, Experiment design (**d**), Oil Red O staining (**e**) and TG concentration (**f**) of primary mouse hepatocytes treated by the medium from the indicated groups. Scale bar, 50 μm, n = 3 biological samples per group (mean ± SD); at least 3 independent experiments were performed and similar results were obtained. Images were taken under an Olympus BX60 microscope. $^{**}P_{TG\ (Vec+CT\ vs\ Vec+PA)} = 0.0025$, $^{***}P_{TG\ (Vec+PA\ vs\ UTX\ +PA)} = 0.0282$ (one-way ANOVA). Source data are provided in the Source Data file.

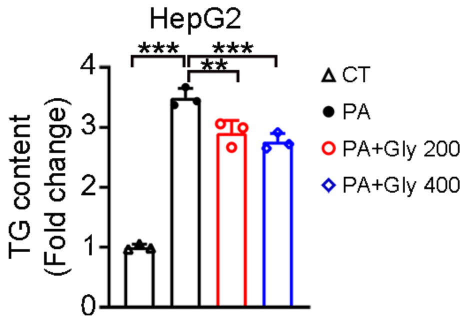

Supplementary Fig. 17 Glycine treatment downregulated triglyceride level in palmitic acid treated HepG2 cells. n = 3 biological samples per group (mean ± SD); at least 3 independent experiments were performed and similar results were obtained. $^{***}P_{TG\ (CT\ vs\ PA)} < 0.0001$, $^{**}P_{TG\ (PA\ vs\ PA+Gly200)} = 0.0035$; $^{***}P_{TG\ (PA\ vs\ PA+Gly400)} = 0.0009$ (one-way ANOVA). Source data are provided in the Source Data file.

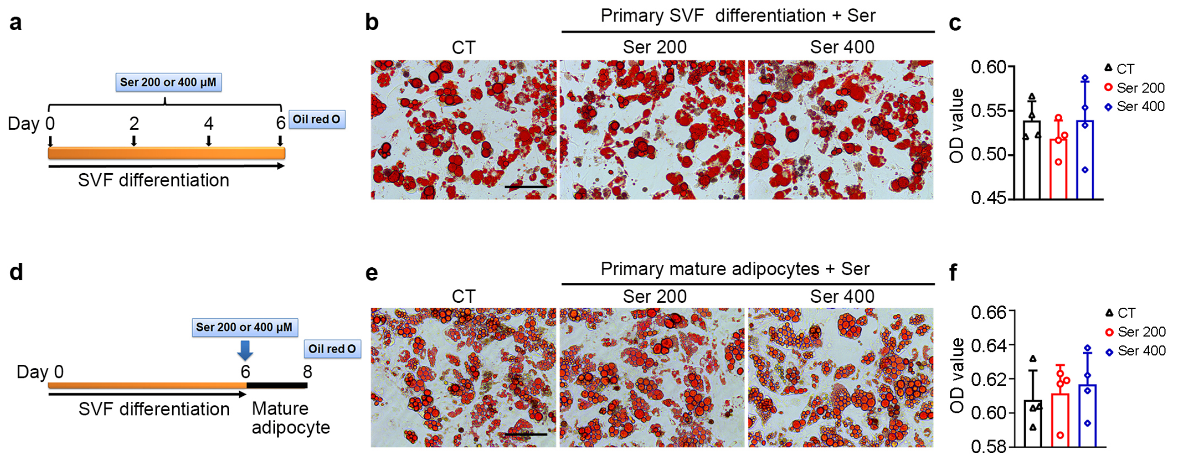

Supplementary Fig. 18 Serine treatment did not affect adipocyte differentiation or lipid accumulation in mouse primary adipocytes. a-c, Experimental design (**a**), Oil Red O staining (**b**) and lipid accumulation (**c**) for serine treated primary SVF cells from adipocyte differentiation Day 0. n = 4 biological samples per group (mean ± SD); at least 3 independent experiments were performed with similar results. **d-f**, Experimental design (**d**), Oil Red O staining (**e**) and lipid accumulation (**f**) for serine treated matured adipocytes differentiated from primary SVF cells. n = 4 biological samples per group (mean ± SD); at least 3 independent experiments were performed with similar results. Scale bar, 50 μm. Source data are provided in the Source Data file.

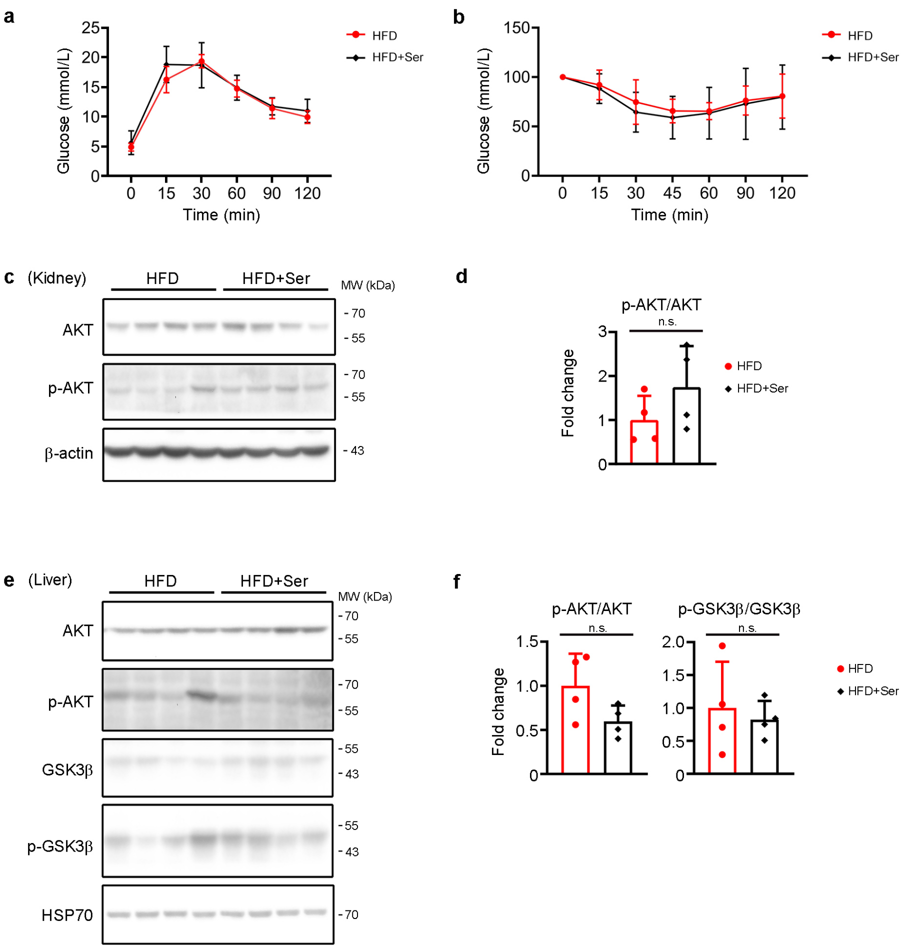

Supplementary Fig. 19 Serine treatment showed no obvious effect on insulin signaling pathway in the liver and kidney of HFD-fed mice. a-b, Glucose tolerance test (GTT) and insulin tolerance test (ITT) results of indicated groups. a, n = 6, 5 independent animals for HFD or HFD+Ser group, respectively; b, n = 4 independent animals (mean ± SD, unpaired, two-tailed t-test). **c-d**, Representative Western blots with densitometric quantitative results of p-AKT/AKT in the kidney of serine treated HFD-fed mice. n = 4 independent animals (mean ± SD, unpaired, two-tailed t-test). **e-f**, Representative Western blots with densitometric quantitative results of p-AKT/AKT, p-GSK3β/GSK3β in the liver of serine treated HFD-fed mice. n = 4 independent animals (mean ± SD, unpaired, two-tailed t-test). Source data are provided in the Source Data file.

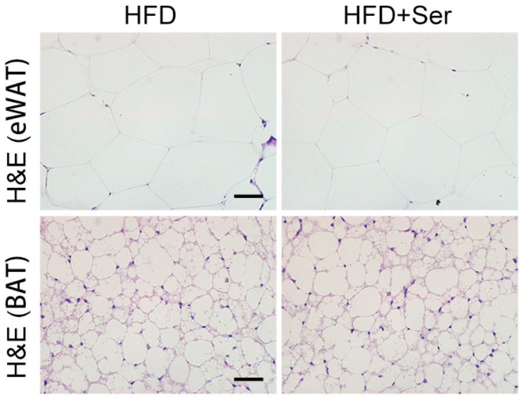

Supplementary Fig. 20 Serine treatment showed no obvious effect on adipocyte hypertrophy under HFD stress. Representative H&E staining in the adipose tissues of HFD-fed mice with serine treatment. n = 4 independent animals. Scale bar, 50 μm.

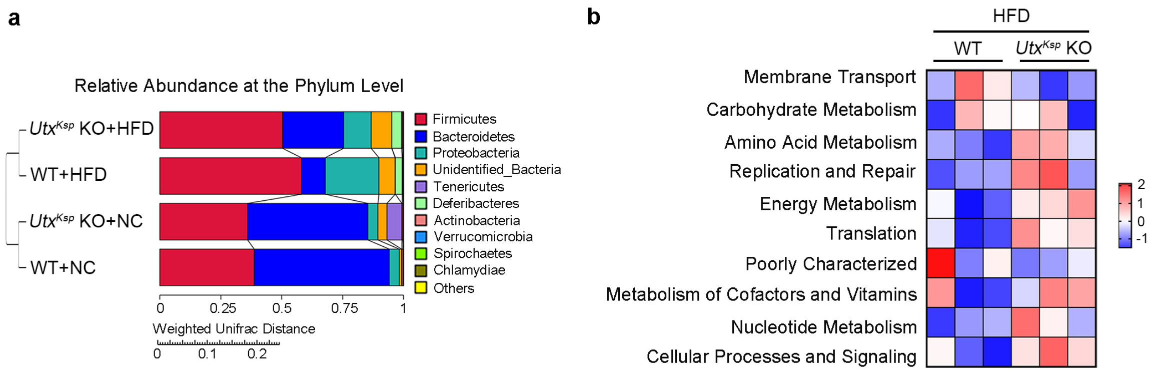

Supplementary Fig. 21 Fecal metagenomic analysis of *Utx*^{Ksp} KO mouse under HFD stress. a, Relative phylum abundance in fecal samples between groups. The 10 most abundant taxa are shown at the phylum level. **b**, PICRUSt prediction of functional profiling of the microbial communities based on the 16S rRNA gene sequences. Each biological sample were obtained from 2-3 mice, and each group contains 3 biological samples. WT+NC, wild-type mice fed with normal chow; *Utx*^{Ksp} KO+NC, *Utx*^{Ksp} KO mice fed with normal chow; WT+HFD, wild-type mice fed with high fat diet; *Utx*^{Ksp} KO+HFD, *Utx*^{Ksp} KO mice fed with high fat diet.

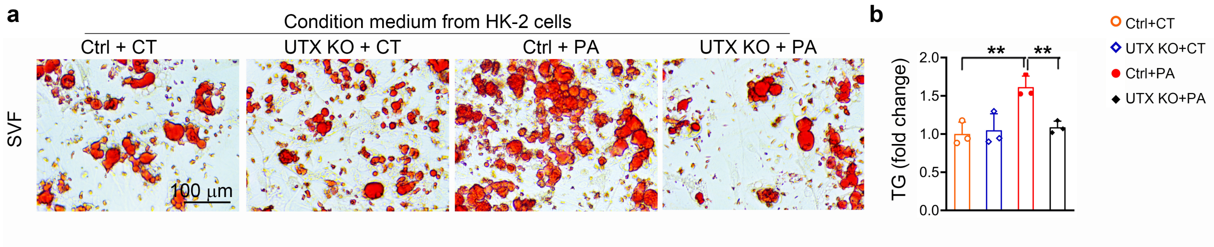

Supplementary Fig. 22 Conditional medium from UTX knockout HK-2 cells altered lipid accumulation in palmitic acid treated primary mouse SVF cells. Representative images of Oil Red O staining (**a**) and TG concentration (**b**) of primary mouse SVF cells treated by the medium from indicated groups, n = 3 biological samples per group (mean ± SD); at least 3 independent experiments were performed and similar results were obtained. $^{**}P_{TG\ (Ctrl+CT\ vs\ Ctrl+PA)} = 0.0037$, $^{**}P_{TG\ (Ctrl+PA\ vs\ UTX\ KO+PA)} = 0.0089$ (one-way ANOVA). Scale bar, 100 μm. Source data are provided in the Source Data file.

Supplementary Table 1. Characteristics of subjects with obesity and the controls whose renal sections used for immunohistochemical study.

Group	Patient ID	Age (year)	Gender	BMI (kg/m^2)	CREA (μmol/L)	eGFR (mL/min/ $1.73m^2$)	BUN (mmol/L)	Alb (g/L)	Globin (g/L)	TG (mmol/L)	TC (mmol/L)	Proteinure (g/24 h)	Blood pressure (mmHg)	Smoking (cigarette/day)	Alcohol (mL/day)	Diabetes history (year)
obesity	1	53	Female	30	55.5	105.4	4.7	39.7	N.A.	1.2	4.2	0.6	119/79	None	None	None
	2	22	Male	31	63.2	146.2	3.3	45	24	1.7	4.6	N.A.	119/76	15	None	None
	3	30	Male	30	53.2	167.5	2.6	42.4	18.2	1.8	2.61	0.4	N.A.	None	None	None
	4	44	Female	29	54.5	111.8	4.1	43.1	23.8	2.2	N.A.	N.A.	180/101	None	None	None
	5	29	Male	29	67.4	128.4	4.2	52.7	23.4	5.1	5.1	N.A.	142/104	None	None	None
control	1	55	Female	22	92	58.4	3.9	40.5	26.1	1.0	3.76	1.4	130/80	None	None	None
	2	49	Female	23	52.9	113.2	4.5	19.8	19.1	3.7	9.7	0.5	120/85	None	None	None
	3	38	Male	23	71.6	113.3	5.2	43	24.7	1.8	5.2	0.3	131/95	None	None	None
	4	30	Female	19	55	119.6	4.8	16.2	18.9	1.6	10.6	N.A.	129/73	None	None	None

Control: normal weight, BMI < 23 kg/m^2; obesity: BMI ≥ 27.5 kg/m^2 (according to Hsu *et al.*, *Diabetes Care* 38, 150-158, 2015). Abbreviations: N.A., not available. BMI, body mass index; CREA, serum creatinine; eGFR, estimated glomerular filtration rate; BUN, blood urea nitrogen; Alb, albumin; TG, triglyceride; TC, total cholesterol. Drug usage history unavailable. Source data are provided in the Source Data file.

Supplementary Table 2. Tissue weights of WT and *Utx*^{Ksp} KO mice fed with NC or HFD.

	WT+NC	*Utx*^{Ksp} KO+NC	WT+HFD	*Utx*^{Ksp} KO+HFD
Kidney weight (g)	0.20 ± 0.02	0.20 ± 0.03	0.22 ± 0.03	0.21 ± 0.02
Liver weight (g)	1.48 ± 0. 07	1.35 ± 0.06	1.74 ± 0.43	1.21 ± 0.22$^{\#}$
eWAT weight (g)	0.33 ± 0.06	0.60 ± 0.12	2.26 ± 0.32***	1.88 ± 0.72
iWAT weight (g)	0.21 ± 0.03	0.37 ± 0.07	2.12 ± 0.46***	1.14 ± 0.54$^{\#\#\#}$
BAT weight (g)	0.12 ± 0.01	0.16 ± 0.03	0.57 ± 0.10***	0.33 ± 0.12$^{\#\#\#}$

Data are presented as mean ± SD. Statistical significance was determined by one-way ANOVA. NC, normal chow; HFD, high fat diet. Source data are provided in the Source Data file. $^{***}P_{eWAT\ (WT+NC\ vs\ WT+HFD)} < 0.0001$, $^{***}P_{iWAT\ (WT+NC\ vs\ WT+HFD)} < 0.0001$, $^{***}P_{BAT\ (WT+NC\ vs\ WT+HFD)} < 0.0001$; $^{\#}P_{Liver\ (WT+HFD\ vs\ Utx^{Ksp}\ KO\ +HFD)} = 0.0112$, $^{\#\#\#}P_{iWAT\ (WT+HFD\ vs\ Utx^{Ksp}\ KO\ +HFD)} = 0.0005$; $^{\#\#\#}P_{BAT\ (WT+HFD\ vs\ Utx^{Ksp}\ KO\ +HFD)} = 0.0003$.

Supplementary Table 3. Tissue weights of WT and *Utx*^{Pax2} KO mice fed with NC or HFD.

	WT+NC	*Utx*^{Pax2} KO+NC	WT+HFD	*Utx*^{Pax2} KO+HFD
Kidney weight (g)	0.21 ± 0.02	0.15 ± 0.02	0.25 ± 0.02**	0.15 ± 0.01$^{\#\#\#}$
Liver weight (g)	1.37 ± 0.10	1.45 ± 0.32	1.99 ± 0.54*	1.22 ± 0.22$^{\#\#}$
iWAT weight (g)	0.27 ± 0.08	0.15 ± 0.04	1.63 ± 0.54***	1.37 ± 0.48
eWAT weight (g)	0.41 ± 0.13	0.38 ± 0.09	1.59 ± 0.30***	0.86 ± 0.19$^{\#\#\#}$
BAT weight (g)	0.16 ± 0.02	0.14 ± 0.04	0.51 ± 0.10***	0.26 ± 0.09$^{\#\#\#}$

Data are presented as mean ± SD. Statistical significance was determined by one-way ANOVA. NC, normal chow; HFD, high fat diet. Source data are provided in the Source Data file. $^{**}P_{Kidney\ (WT+NC\ vs\ WT+HFD)} = 0.0040$, $^{*}P_{Liver\ (WT+NC\ vs\ WT+HFD)} = 0.0237$, $^{***}P_{iWAT\ (WT+NC\ vs\ WT+HFD)} < 0.0001$, $^{***}P_{eWAT\ (WT+NC\ vs\ WT+HFD)} < 0.0001$, $^{***}P_{BAT\ (WT+NC\ vs\ WT+HFD)} < 0.0001$; $^{\#\#\#}P_{Kidney\ (WT+HFD\ vs\ Utx^{Pax2}\ KO\ +HFD)} < 0.0001$, $^{\#\#}P_{Liver\ (WT+HFD\ vs\ Utx^{Pax2}\ KO\ +HFD)} = 0.0049$, $^{\#\#\#}P_{eWAT\ (WT+HFD\ vs\ Utx^{Pax2}\ KO\ +HFD)} < 0.0001$, $^{\#\#\#}P_{BAT\ (WT+HFD\ vs\ Utx^{Pax2}\ KO\ +HFD)} = 0.0002$.

Supplementary Table 4. Characteristics of subjects with diabetic kidney disease and the controls whose renal sections used for immunohistochemical study.

**Group**	**Patient ID**	**Age (year)**	**Gender**	**Blood glucose (mmol/L)**	**HbA1c**	**eGFR (mL/min/ 1.73m^2)**	**CREA (μmol/L)**	**BUN (mmol/L)**	**Alb (g/L)**	**Globin (g/L)**	**TG (mmol/L)**	**TC (mmol/L)**	**Proteinure (g/24 h)**	**Blood pressure (mmHg)**	**Smoking (cigarett e/day)**	**Alcohol (mL/day)**	**Diabetes history (year)**
	1	67	Male	7.6	N.A.	35.2	178.7	N.A.	N.A.	N.A.	N.A.	N.A.	7.2	140/70	None	None	12
	2	60	Female	8.9	N.A.	93.8	60.1	N.A.	N.A.	N.A.	N.A.	N.A.	5.7	180/100	None	None	5
	3	50	Male	5.9	6.1%	63.9	114	7.5	26.5	26.1	2.2	4.8	9.9	151/85	None	None	16
	4	58	Female	9.6	N.A.	30.7	159.2	N.A.	N.A.	N.A.	N.A.	N.A.	1.9	160/90	None	None	7
**DKD**	5	48	Female	10.9	N.A.	99.1	59.6	N.A.	N.A.	N.A.	N.A.	N.A.	3.4	160/100	None	None	3
	6	57	Female	7.2	7.5%	3.7	926	5.9	35.5	30.3	3.5	8.1	2.6	139/81	None	None	7
	7	73	Male	N.A.	6.6%	59.2	112.2	N.A.	31.8	N.A.	N.A.	N.A.	3.9	160/90	None	None	15
	8	51	Male	N.A.	11%	39.8	165.6	7.01	122.7	75.5	1.8	8.8	N.A.	130/89	None	None	10
	9	27	Female	N.A.	7.4%	19.8	266.3	16.23	26.8	27.5	1.2	5.9	3.6	N.A.	None	None	N.A.
	1	38	Male	4.7	N.A.	107.2	80	4.8	29.2	18	0.9	3.5	2.3	109/65	None	None	None
	2	63	Female	N.A.	N.A.	92.4	60.4	4.9	19.4	20.1	4.5	7.8	6.7	149/83	None	None	None
	3	55	Male	6.1	N.A.	123.2	62.4	N.A.	N.A.	N.A.	N.A.	N.A.	2.8	130/80	None	None	None
	4	26	Male	5.1	N.A.	108.3	79.6	N.A.	N.A.	N.A.	N.A.	N.A.	11.4	124/80	10-15	50	None
**control**	5	42	Female	5.8	N.A.	144.2	44.1	N.A.	N.A.	N.A.	N.A.	N.A.	5.0	110/70	None	None	None
	6	59	Female	6.4	N.A.	55.5	95	N.A.	N.A.	N.A.	N.A.	N.A.	3.0	120/76	None	None	None
	7	35	Male	N.A.	5.5%	115.1	71.7	3.6	22.1	17.7	2.6	7.8	7.5	115/82	None	None	None
	8	58	Male	4.9	N.A.	76.4	80.7	6	29.9	25.2	1.6	5.0	N.A.	N.A.	None	None	None
	9	46	Female	5.4	N.A.	139	44.8	2.7	31.1	24.9	1.5	4.3	0.5	134/94	None	None	None

DKD, diabetic kidney disease; control, membrane nephropathy, N.A., not available. Hb1Ac, hemoglobin A1c; eGFR, estimated glomerular

filtration rate; CREA, serum creatinine; BUN, blood urea nitrogen; Alb, albumin; TC, total cholesterol; TG, triglyceride. Drug usage history unavailable. Source data are provided in the Source Data file.

Supplementary Table 5. Characteristics of clinical serum samples used in this study

Group	Age (year)	Gender (M/F)	Body weight (kg)	BMI (kg/m^2)	CREA (μmol/L)	BUN (mmol/L)	Alb (g/L)	Globin (g/L)	TG (mmol/L)	TC (mmol/L)
**CT** (n = 39)	41.2 ± 16.6	19/20	60.4 ± 7.1	21.6 ± 1.6	67.3 ± 12.8	5.0 ± 1.6	41.9 ± 4.1	28.4 ± 3.6	1.3 ± 0.6	4.7 ± 1.0
**Overweight** (n = 23)	51.9 ± 18.7	14/9	74.5 ± 6.3	26.2 ± 0.7	102.9 ± 96.8	6.7 ± 4.5	39.6 ± 4.5	25.6 ± 3.1	1.7 ± 1.0	4.1 ± 0.8
**Obesity** (n = 24)	47.0 ± 15.3	15/9	86.6 ± 13.4	31.0 ± 2.6	68.8 ± 16.5	4.9 ± 1.4	40.1 ± 3.4	26.3 ± 4.0	2.3 ± 1.7	4.5 ± 1.2
**Obesity-related renal dysfunction** (n = 12)	58.3 ± 9.6	11/1	80.7 ± 6.2	29.1 ± 1.5	448.3 ± 367.3	18.0 ± 10.8	37.6 ± 2.6	27.7 ± 5.5	2.1 ± 1.2	3.6 ± 0.9

CT: individuals with normal body weight (BMI < 23 kg/m^2); overweight: subjects whose 23 ≤ BMI < 27.5 kg/m^2; obesity: subjects whose BMI ≥ 27.5 kg/m^2 without renal dysfunction; obesity related renal dysfunction: subjects whose BMI ≥ 27.5 kg/m^2 with renal dysfunction. Abbreviations: M, male; F, female; BMI, body mass index; CREA, serum creatinine; BUN, blood urea nitrogen; Alb, albumin; TG, triglyceride; TC, total cholesterol. Some subjects with no available albumin and globin levels. Drug usage history unavailable. Source data are provided in the Source Data file.

Supplementary Table 6. Tissue weights of HFD-fed C57BL/6 mice with/without serine treatment.

	HFD	HFD + Ser
Kidney weight (g)	0.22 ± 0.04	0.20 ± 0.02
Liver weight (g)	2.09 ± 0.40	1.57 ± 0.37*
eWAT weight (g)	2.33 ± 0.53	2.31 ± 0.74
iWAT weight (g)	2.34 ± 0.28	1.88 ± 0.72
BAT weight (g)	0.47 ± 0.08	0.43 ± 0.14

Data are presented as mean ± SD. Statistical significance was determined by two-tailed Student's *t* test. Source data are provided in the Source Data file. $^{*}P_{Liver\ (HFD\ vs\ HFD+Ser)}$ = 0.0261.

Supplementary Table 7. Primer sequences used for genotyping.

Utx-flox	GGTCACTTCAACCTCTTATTGGA	ACGAGTGATTGGTCTAATTTGG
Ksp-Cre	GCAGATCTGGCTCTCCAAAG	AGGCAAATTTTGGTGTACGG
Pax2-Cre	TCAAATGGCTCTCCTCAAGC	AGCTGGCCCAAATGTTGCTG
Alb-Cre	GAACCTGATGGACATGTTCAGG	AGTGCGTTCGAACGCTAGAGCCTGT
Adi-Cre	ACG GACAGAAGCATTTTCCA	GGATGTGCCATGTGAGTCTG

Supplementary Table 8. Antibodies used in the present study.

**Antibody**	**Catalog number**	**Company**
β-actin	A5316	Sigma Aldrich
UTX	33510 (for WB and IF)	Cell Signaling Technology
UTX	ab36938 (for IHC)	Abcam Biochemicals
FLAG	F1804	Sigma Aldrich
RNF114	HPA021184	Sigma Aldrich
PHGDH	14719-1-AP	Proteintech
HA	H9658	Sigma Aldrich
H3K27me3	PTM-622 (for WB)	PTM Biolabs
H3K27me3	ab6002 (for ChIP)	Abcam Biochemicals
H3	9715	Cell Signaling Technology
AKT	4691	Cell Signaling Technology
p-AKT	9271s	Cell Signaling Technology
SGLT2	24654-1-AP	Proteintech
LDLR	A14996	ABclonal
FXR	Sc-25309	Santa Cruz
PPARα	Sc-9000	Santa Cruz
SREBP1	Sc-367	Santa Cruz
p-GSK 3β	9323s	Cell Signaling Technology
GSK 3β	9832S	Cell Signaling Technology
α-SMA	A2547	Sigma Aldrich
WT1	Sc-393498	Santa Cruz

Supplementary Table 9. Primers for plasmids constructed in the study.

Primer name	Sequence (5'-3')
FLAG-PHGDH WT	Forward: GCGGTCGACCATGGCTTTTGCAAATCTGCGG
	Reverse: ACGAGCGGCCGCGAAGTGGAACTGGAAGGCTTC
PHGDH K146R	Forward: GAGCTGAATGGAAGGACCCTGGGAATT
	Reverse: AATTCCCAGGGTCCTTCCATTCAGCTC
PHGDH K289R	Forward: GGTGCCAGCACCAGGGAGGCTCAGAGC
	Reverse: GCTCTGAGCCTCCCTGGTGCTGGCACC
PHGDH K310R	Forward: CATGGTGAAGGGGAGATCTCTCACGGGGG
	Reverse: CCCCCGTGAGAGATCTCCCCTCACCATG
PHGDH K330R	Forward: CTCTCCACACACCAGGCCTTGGATTGGTCTG
	Reverse: GACCAATCCAAGGCCTGGTGTGTGGAGAGAAG
PHGDH K364R	Forward: CAGGGAACATCCCTGAGGAATGCT GGGAACTG
	Reverse: CAGTTCCCAGCATTCCTCAGGGATGTT CCCTG
PHGDH K384R	Forward: AAAGAGGCTTCCAGGCAGGCGGATGTG
	Reverse: CACATCCGCCTGCCTGGAAGCCTCTTT
HA-RNF114	Forward: GCGGTCGACCATGGCGGCGCAACAGCGGGAC
	Reverse: ACGAGCGGCCGCTCACTGGTCGATGATGGAGCGCTG
pGL3-basic-CIDEA	Forward: ATTACGCGTCCACCCCACCCCCATGTCCAC
	Reverse: ATTAGATCTGCGCCCAGCTCCCGTTGTGATT
pGL3-basic-CIDEC	Forward: ATTACGCGTGGCGTGAGCCACGGCACCCAG
	Reverse: ATTAGATCTCCTGAATCAGAATCTGCACTTGA

Supplementary Table 10. qPCR primers used in the present study.

Gene	Forward primer	Reverse primer
M/H/R *Rn18s*	TTAAGAGGGACGGCCGGGGG	GCCGGGTGAGGTTTCCCGTG
M Actb	GCTCTTTTCCAGCCTTCCTT	CGGATGTCAACGTCACACTT
H ACTB	TGGACTTCGAGCAAGAGATG	GAAGGAAGGCTGGAAGAGTG
M *Utx*	TGGAGGATCTGATGCAAGTCT	ATCAAGATGAGGCGGATGGT
H *UTX*	GCTGGAACAGCTGGAAAGTC	GAGTCAACTGTTGGCCCATT
H *MOGAT1*	AGTGTTGGGCTGGTTTCAGT	AACAAATCCTTTCCGCTGGC
H *GPAM*	TGAACAGATAGCACTGGGGC	AAAGCCAGGATCATTGGGGC
H *CIDEA*	CTCATCAGGCCCCTGACATT	ATGGTTGGAGACCCGGAAAG
H *CIDEC*	TGAGAAACATGGAGTCCAACGC	TGGGGTAGAGAAGGCTAAGGG
M *Mogat1*	CCAGCGCAAAGGGTTTGTT	CACCAAAAGAAAATACTGGAACCA
M *Cidea*	CTTGGGGGTGGTACCCAGTG	ATCCACGCAGTTCCCACACA
M *Cidec*	GCTGAAGGGGCAGAAGTGGA	GCGCTTGGCCTTGTAGCAGT
M *Gpam*	GCCAGCAAGTCCTGCGCTAT	CCTGCTCGTGTGGGTGATTG
M *Phgdh*	AGTGGACCACGAGAATGTCA	CCTTCACCATGTCCACAAAC
M *Scd1*	TTCTCAGAAACACACGCCGA	AGCTTCTCGGCTTTCAGGTC
M *Srebp1*	AAGACAGATGCAGGAGCCAC	ATGGTCCCTCCACTCACCAG
M *Acly*	GGCCAGAGAGCTGGGTTTGA	CCCGAGCACAGATGATGGTG
M *Fasn*	CCTGGCTGCCTACTACATCG	CACATTTCAAAGGCCACGCA
M *Fxr*	GGCTGAATGTATGTATACAGGTTTG	CAGCGTGCTGCTTCACATTT
M *Ppara*	TGACGTTTGTGGCTGGTCAA	CAGATGGGGCTCTCTGTGTC
M *Ldlr*	CCAATCGACTCACGGGTTCA	CTCACACCAGTTCACCCCTC
M *Cd36*	TTGGCCAAGCTATTGCGACA	CTGGAGGGGTGATGCAAAGG
M *Vldlr*	TCAGTCCCAGGCAGCGTAT	CTTGATCTTGGCGGGTGTT
M *Slc27a1*	GGGAGCCTGACACCCCTCTT	CCCCTGGACACTGGTCCAAC
M *Acox1*	GGGAGTGCTACGGGTTACATG	CCGATATCCCCAACAGTGATG

M *Cpt1a*	CCATGATGGACCCCACAACA	TGGTCAACCTCCATGGCTCA
M *Cpt1b*	ATCTTGGTGGCATGGCTGGT	GGGACTGGTCGATTGCATCC
M *Glut4*	ACTCATTCTTGGACGGTTCC	TAGCTGTGCCCAGCATAGAC
M *Sglt2*	CATTGGTGTTGGCTTGTGGTC	AAATGACCGCTGCCGATGTT
M *Sglt1*	GTCGTCACCGTCTTGGTCAT	GTAGACTCCAGCACAGACGG
M *G6pc*	CAGTGGTCGGAGACTGGTTC	GTCCAGGACCCACCAATACG
M *Pepck*	TGAAAGGCCGCACCATGTAT	GGGCGAGTCTGTCAGTTCAA
M *Pc*	CTGCAGCAAGTTTGGTTGCG	TAGATGTTAGCTCCGCCCTG
M *Fbp1*	GCACAGCTCTATGGTATCGCT	CACAGGTAGCGTAGGACGAC
M *Gapdh*	ACCCTTAAGAGGGATGCTGC	CGGGACGAGGAAACACTCTC
M *Pgk*	GGCATTCTGCACGCTTCAAA	CGACATTTTGGCAACACCGT
M *Pgam*	CGCCTCAATGAGCGACACTA	TCACCATGCTTAGCAGCAGT
M *Aldoa*	CCTAGCCGCGTTCGCTC	GACAGGCGGGTCATGTTGAAG
M *Gpi*	GACACCCTTCATTCTGGGGG	TCCCACATGATGCCCTGAAC
H *ASCT1*	TCTCCTCGCCTTTCTCGCAC	AAAGACGGGGTTCCCAATGA
H *ASCT2*	GTAACCGCTACTCCCGGACA	CAGGGGACCCAGGCTCTTAG

Supplementary Table 11. Primer sequences used for ChIP assay.

Gene	Forward primer	Reverse primer
M *Cidea* p1	CCTGTTAGGACACTCCGCTC	GGGGTGACTGGTGACATCAT
M *Cidea* p2	AACAAGCGAATCCATCAGAGC	ACAGGGTATCGGAGTGACCA
M *Cidea* p3	TGCTGGGAGGAGAGACACAA	GGCCTCCAAGCTCACAGATA
M *Cidea* p4	CAAGGGGCTCCCTTTGTCTT	TGGGGTGAGAGTCTGGAGAG
M *Cidec* p1	TTCCCCATGCTCTTTTCCCC	CCCAGGCTTCCCTCCATTTT
M *Cidec* p2	GCTCAGGCTTGTCTTGAATTAGA	GGGGTGGGAAATCACAAAAGTT
M *Cidec* p3	ACCTTTAGTCCCGGCTCTCT	AGGGATCTGTCACCTCGTCA
M *Cidec* p4	AGGCCGTCTTGCTTTCTGATG	GCGACATTCCTTCATCGAGT
